# Osteocalcin has a muscle-protective effect during weight loss in men without metabolic syndrome: a multicenter, prospective, observational study

**DOI:** 10.3389/fendo.2023.1308452

**Published:** 2023-11-29

**Authors:** Yi Xiang, Wenyi Lu, Xiaomeng Mao, Jing Zou, Jialu Wang, Renying Xu, Qingya Tang

**Affiliations:** ^1^ Xinhua Hospital, School of Medicine, Shanghai Jiao Tong University, Shanghai, China; ^2^ Renji Hospital, School of Medicine, Shanghai Jiao Tong University, Shanghai, China

**Keywords:** weight loss, high-protein, energy-restricted diets, metabolic syndrome, osteocalcin, muscle mass

## Abstract

**Objective:**

Weight reduction often accompanies muscle loss. Existing studies highlight the involvement of osteocalcin (OC) in energy metabolism and its potential to prevent age-related muscle loss. Nevertheless, these studies predominantly involve individuals with hyperglycemia, yielding conflicting research outcomes. This study investigated the protective role of OC against muscle loss during weight reduction in individuals without metabolic syndrome (MetS).

**Measures:**

We enrolled 130 overweight or obese individuals without MetS in a 4-month high-protein, energy-restricted dietary weight management program conducted at two clinic centers. Body composition and laboratory tests were assessed both before and after weight loss. Correlation and regression analysis were made between the changes in metabolic indicators and muscle mass during weight loss.

**Results:**

Following weight loss, there was a decrease in body mass index (BMI), percentage of body fat (PBF), visceral fat area (VFA), fasting insulin (FINS), homeostasis model assessment insulin resistance (HOMA-IR), glycated haemoglobin (HbA1c), and lipid profile, and increase in the percentage of skeletal muscle (PSM) and vitamin D. There was no change in osteocalcin (OC) during the intervention. Correlation analysis of the relative changes in all metabolic indicators revealed a positive correlation between OC and PSM (r=0.383, p=0.002). Multiple linear regression analysis found that OC has a significant protective effect on muscles during weight loss in males after adjusting for confounding factors (β=0.089, p=0.017).

**Conclusion:**

High-protein, energy-restricted diets demonstrate efficacy in enhancing metabolic indicators within the weight-loss population. Furthermore, OC exhibits a protective effect on muscle mass during weight reduction in individuals without MetS, with this effect being particularly evident in males.

## Introduction

With the amelioration of living standards, the incidence of overweight and obesity is on the rise. Unaddressed, this trend is conducive to the development of metabolic syndrome (MetS), which in turn can precipitate conditions such as diabetes mellitus (DM) and cardiovascular diseases, escalating the susceptibility to various other maladies (1, 2). The fundamental tenet of weight loss revolves around diminishing energy intake and augmenting energy expenditure. However, prolonged inadequate nutritional intake risks diminishing muscle mass and basal metabolic rate (BMR), intensifying challenges in the later phases of weight loss and elevating the likelihood of weight regain (3). Consequently, a balanced, nutritious diet and the preservation of muscle mass constitute the central requisites for scientifically sound weight loss.

Osteocalcin (OC), a noncollagenous acidic glycoprotein originating from osteoblasts, traditionally served as a marker for bone formation and has recently garnered recognition for its endocrine regulatory properties (4, 5). While OC research has predominantly been conducted in murine models, disparate outcomes have been observed in different mouse strains. Studies led by Karsenty’s group have substantiated OC’s hormonal role in regulating pancreatic insulin secretion, testicular testosterone synthesis, and muscle mass (6, 7). Nonetheless, specific independent mouse experiments failed to replicate these hormonal functions, thereby casting doubt on the hormonal regulatory effects of OC (8–10).

Human-level investigations into the hormonal regulation of OC primarily center on insulin sensitivity, diabetes risk, and lipid metabolism (11, 12). Limited studies have explicitly explored the correlation between OC and muscle mass (13, 14). However, most of these inquiries were grounded in populations with MetS, positing that OC indirectly modulates muscle mass by regulating glucose metabolism (14–18).

We assembled a cohort of individuals who had not yet manifested MetS to address these gaps. They partook in a 4-month high-protein, energy-restricted dietary weight management initiative, which involved comprehensive assessments of body composition and metabolic indicators, both pre-and post-weight loss. This study aimed to scrutinize alterations in these parameters and discern the impact of OC on muscle mass.

## Materials and methods

### Subject population

Participants undergoing weight management were recruited from two affiliated hospitals, Xinhua Hospital and Renji Hospital, associated with Shanghai Jiao Tong University School of Medicine in 2021. A total of 130 individuals were included in a 4-month weight management program.

### Inclusion criteria

1) Generally healthy individuals aged 18 to 65 years;

2) Body mass index (BMI) ranged from 24 to 35 kg/m^2^;

3) Waist circumference exceeding 85 cm for males and 80 cm for females.

### Exclusion criteria

1) Diagnosis of type I or type II DM or fasting blood glucose (FBG) ≥ 7 mmol/L (126 mg/dL) or glycated haemoglobin (HbA1c) ≥ 6.5%;

2) Aspartate aminotransferase (AST) and alanine aminotransferase (ALT) >2.5 times normal (AST: 0-35 units/L or 0-0.58 μKat/L; ALT: 4-36 units/L), low-density lipoprotein-cholesterol (LDL-C) > 1.5 times normal (<130 mg/dL), triglyceride (TG) > 2.2 mmol/L;

3) History of gout or uric acid levels >1.2 times normal (normal values: men, 4.0-8.5 mg/dL or 0.24-0.51 mmol/L; women, 2.7-7.3 mg/dL or 0.16-0.43 mmol/L);

4) With malignant hypertensive crisis or uncontrolled hypertension (systolic blood pressure ≥160 mmHg or diastolic blood pressure ≥100 mm Hg) or with other clinically severe conditions;

5) History of bariatric surgery or usage of diet pills or lipid-lowering drugs.

### Study protocol and methods

All participants in the weight management program adhered to a high-protein, energy-restricted diet. They were mandated to maintain a daily step count of no less than 10,000. The dietary regimen involved a daily protein intake constituting 20% of the total energy intake. The daily energy intake was determined by the basal metabolic rate (BMR), as measured through body composition (Eqn.1).

At the program’s commencement, personalized dietary plans were devised for each participant. Throughout the initial month, individuals were instructed to maintain a dietary diary for three days each week. Our team offered prompt guidance and corrections based on the provided diaries. Participants unable to adhere to the prescribed dietary requirements after four weeks were discontinued from the program. Following adjustments to dietary patterns, regular monthly check-ins were conducted over the subsequent three months to monitor progress and provide ongoing support.

Body composition was measured once a month. Bone density, grip strength, and laboratory tests were conducted at the program’s initiation and conclusion to capture baseline and endpoint data. Step counts were monitored using the 4th generation Xiaomi Smart Band.

Bioelectrical impedance analysis was conducted with the Inbody 720 (Biospace, Seoul, South Korea) to evaluate body composition comprehensively. Measurements encompassed weight, fat mass (FM), percentage of body fat (PBF), lean body mass (LBM), visceral fat area (VFA), fat-free mass (FFM), and skeletal muscle mass (SMM). Participants were positioned upright following bladder emptying to ensure measurement accuracy. The percentage of SMM to body weight (PSM) was computed using Eqn.2.

Quantitative ultrasonography (QUS) was utilized to measure the speed of ultrasound (SOS) at the calcaneus (IEC 601–1 Class II Type BF. IPXO, Sahara Bone Densitometer, Hologic, Bedford, MA). Grip strength assessments were conducted using a consistent grip strength tester for both hands, with the highest recorded value utilized for analysis. Height measurements were performed using a rigid stadiometer.

Fasting blood samples were obtained after a period of 10 ± 2 hours of fasting. The collected blood samples were analyzed for the following parameters:

1. Lipid profile: TC, TG, HDL-C, LDL-C;
2. Liver and renal function: alkaline phosphatase, total bilirubin, direct bilirubin, creatinine, blood urea nitrogen, AST, and ALT;
3. Glucose metabolism indicators: FBG, fasting insulin (FINS), homeostasis model assessment insulin resistance (HOMA-IR, Eqn.3), HbA1c, triglyceride-glucose index (TyG index, Eqn.4);
4. Bone metabolism indicators: OC, parathyroid hormone (PTH), vitamin D, calcium (Ca), phosphorus (P);
5. Urine pregnancy tests were conducted on all female subjects.

### Equations employed in the study

Eqn.1:


f(x)=BMR(kcal)×1.25−500kcal

Eqn.2:


f(x)=SMM(kg)÷weight(kg)×100%

Eqn.3:


f(x)=FBG(mmol/L)×FINS(mU/L)÷22.5

Eqn.4:


f(x)=ln(TG(mg/dL)×FBG(mg/dL)÷2)

The institutional ethics committee of Shanghai Xinhua Hospital and Shanghai Renji Hospital approved this study.

### Statistical analysis

Data were presented as the mean ± standard deviation (SD) or median (interquartile range, 25–75%) for continuous variables and proportion for categorical variables. The paired samples t-test was employed for normally distributed continuous variables of paired samples (pre- vs. post-weight loss), while the Wilcoxon signed-rank test was used for non-normally distributed samples. Pearson or Spearman correlation coefficients were calculated to assess the relationship between muscle mass and metabolic indicators. Multiple linear regression analysis was conducted to ascertain the association between muscles and relevant metabolic indicators. Statistical significance was established at p< 0.05.

The calculations were executed utilizing IBM SPSS Statistics, version 26.0 (IBM Corporation, Armonk, NY, USA). The image was generated using GraphPad Prism, version 9.4.1.

## Results

130 subjects, comprising 38 males and 92 females, completed the weight management program. The mean age of the participants was 33 years. The observed average weight loss was 5 kg per individual (78.4 kg vs. 73.3 kg, p<0.001), with a predominant reduction in FM (27.3 kg vs. 22.6 kg, p<0.001). Despite the program’s emphasis on maintaining muscle mass, a marginal decline was noted in both LBM (44.2 kg vs. 43.2 kg, p<0.001) and SMM (27.6 kg vs. 26.6 kg, p<0.001). However, a noteworthy increase in the PSM was evident (36.1% vs. 37.2%, p<0.001). There was no decrease in grip strength or bone mineral density (BMD) among the subjects (**Table 1**).

**Table 1:** Comparison of changes in body composition and metabolic indicators before and after the intervention.

Variables	4-month weight loss program (n=130)		p-value
	Before	After	
Age (y)	33 (29,38)		
Gender (M/F)	38/92		
Weight (kg)	78.4 ± 10.8	73.3 ± 11.0	**<0.001**
BMI (kg/m2)	27.8 (26.2, 30.9)	25.7 (24.3, 28.7)	**<0.001**
FM (kg)	27.3 (22.8, 32.8)	22.6 (19.8, 27.65)	**<0.001**
PBF (%)	35.8 ± 6.8	32.6 ± 6.8	**<0.001**
PSM (%)	36.1 (33.2, 39.2)	37.2 (35.2, 39.1)	**<0.001**
SMM (kg)	27.6 (25.3, 35.1)	26.6 (24.2, 32.5)	**<0.001**
FFM (kg)	47.1 (43, 56.6)	46.0 (42.5, 55.6)	**<0.001**
LBM (kg)	44.2 (40.9, 53.3)	43.2 (39.9, 52.3)	**<0.001**
VFA (m2)	105.1 ± 27.4	95.8 ± 27.6	**<0.001**
BMC (kg)	3.1 ± 0.5	3.0 ± 0.5	**<0.001**
BMR (kcal)	1446 (1356, 1645)	1416 (1340, 1622)	**<0.001**
SOS (m/s)	1516.7 ± 31.8	1517.1 ± 29.4	0.87
Grip strength (kg)	31.7 ± 9.3	31.4 ± 8.7	0.444
FBG (mmol/L)	5.1 (4.9, 5.4)	5.1 (4.9, 5.3)	0.194
HbA1c (%)	5.4 (5.2, 5.6)	5.3 (5.2, 5.6)	**0.005**
FINS (pmol/L)	69.4 (48.2, 87.7)	52.3 (37.9, 80.5)	**<0.001**
HOMO-IR	2.26 (1.5, 3.1)	1.7 (1.2, 2.7)	**<0.001**
TC (mmol/L)	4.6 ± 0.7	4.4 ± 0.8	**<0.001**
TG (mmol/L)	1.1 (0.8, 1.5)	0.86 (0.63, 1.3)	**<0.001**
HDL-C (mmol/L)	1.2 ± 0.2	1.3 ± 0.2	0.095
LDL-C (mmol/L)	2.9 ± 0.7	2.7 ± 0.7	**0.001**
TyG index	6.9 (6.5, 7.2)	6.6 (6.2, 7.0)	**<0.001**
25OHVD (nmol/L)	40.0 (31.8, 47.3)	43.1 (34.2, 52.0)	**<0.001**
Ca (mmol/L)	2.3 ± 0.1	2.3 ± 0.1	0.537
P (mmol/L)	1.1 ± 0.2	1.1 ± 0.2	0.067
PTH (pmol/L)	4.6 (3.5, 5.8)	4.5 (3.7, 5.9)	0.563
OC (ng/mL)	14.2 (12.3, 18.1)	13.9 (11.5, 17.9)	0.361

Data are presented as the mean ± SD, median (interquartile range), or n (%) as appropriate. P< 0.05 was considered statistically significant.

M/F, male/female; BMI, body mass index; FM, fat mass; PBF, percentage of body fat; SMM, skeletal muscle mass; PSM, percentage of SMM; FFM, fat-free mass; LBM, lean body mass; VFA, visceral fat area; BMC, bone mineral content; BMR, basal metabolic rate; SOS, speed of ultrasound; FBG, fasting blood glucose; HbA1c, glycated haemoglobin; FINS, fasting insulin; HOMA-IR, homeostasis model assessment of insulin resistance; TC, total cholesterol; TG, triglycerides; HDL-C, high-density lipoprotein cholesterol; LDL-C, low-density lipoprotein cholesterol; TyG index, triglyceride-glucose index; 25OHVD, 25-OH Vitamin D; Ca, calcium; P, phosphorus; PTH, parathyroid hormone; OC, osteocalcin.

Bold values indicate statistically significant.

Regarding metabolic indicators, although subjects’ FBG did not change throughout the program, HbA1c (5.4% vs. 5.3%, p=0.005), FINS (69.4 pmol/L vs. 52.3 pmol/L, p<0.001), HOMO-IR (2.26 vs. 1.7, p<0.001), TyG index (6.9 vs. 6.6, p<0.001) and the lipid profile all improved significantly. In addition, there was an increase in vitamin D (25OHVD) in the subjects after adjusting for seasonal changes. There were no significant changes in Ca, P, PTH, or OC in the subjects (**Table 1**).

**Figure 1:**
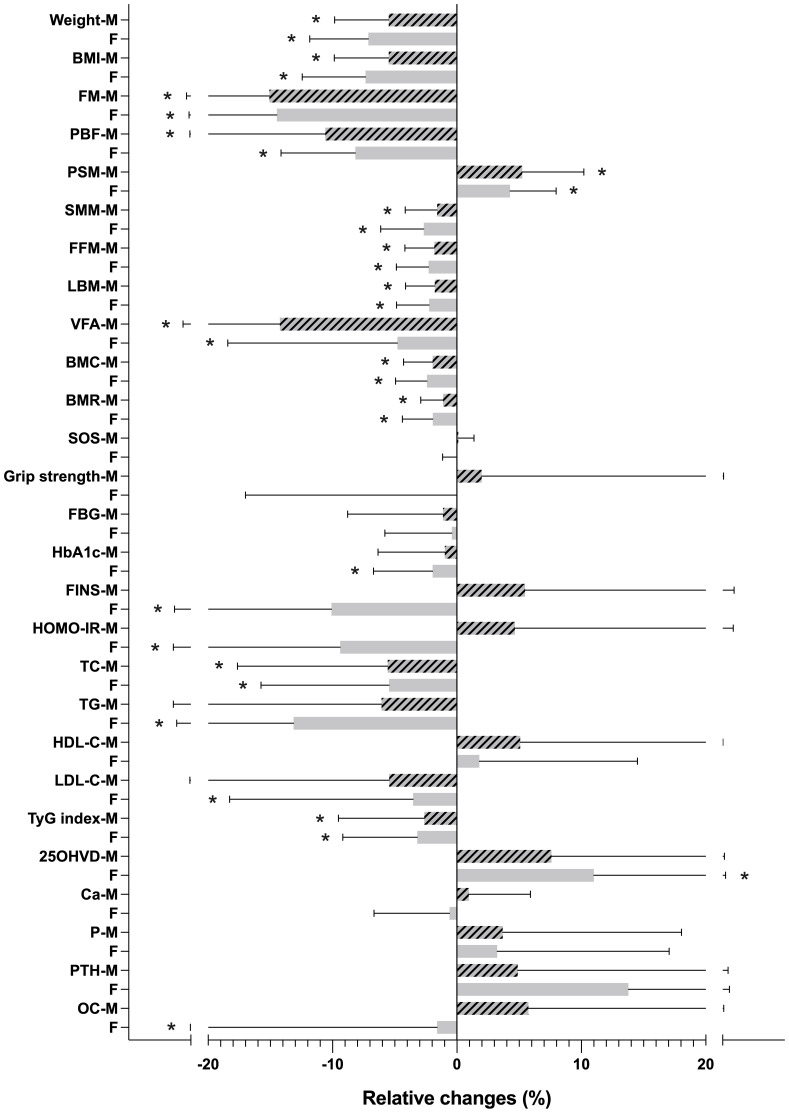
Relative changes (%) in body composition and metabolic indicators during the intervention. *p <  0.05. M, male; F, female; BMI, body mass index; FM, fat mass; PBF, percentage of body fat; SMM, skeletal muscle mass; PSM, percentage of SMM; FFM, fat-free mass; LBM, lean body mass; VFA, visceral fat area; BMC, bone mineral content; BMR, basal metabolic rate; SOS, speed of ultrasound; FBG, fasting blood glucose; HbA1c, glycated haemoglobin; FINS, fasting insulin; HOMA-IR, homeostasis model assessment of insulin resistance; TC, total cholesterol; TG, triglycerides; HDL-C, high-density lipoprotein cholesterol; LDL-C, low-density lipoprotein cholesterol; TyG index, triglyceride-glucose index; 25OHVD, 25-OH Vitamin D; Ca, calcium; P, phosphorus; PTH, parathyroid hormone; OC, osteocalcin.

In the male-female subgroup comparisons of the relative changes during the intervention of each indicator, women exhibited more pronounced reductions in weight and BMI; however, the decrement in SMM was also more significant. Conversely, men experienced greater declines in FM, PBF, and VFA, accompanied by higher increments in PSM. Regarding metabolic indicators, females displayed significant glucose and lipid metabolism enhancements, whereas males did not exhibit notable improvements in HbA1c, FINS, HOMA-IR, TG, and LDL-C. Regarding bone metabolism indicators, only women demonstrated a noteworthy increase in 25OHVD levels (**Figure 1**).

We calculated the correlations between the changes in PSM and all the metabolic indicators during weight loss. The strongest correlation identified was between changes in PSM and OC (r=0.383, p=0.002). Subsequent gender-stratified analysis sustained this correlation, with males exhibiting a correlation coefficient of r=0.480 (p=0.032) and females registering a coefficient of r=0.408 (p=0.009) (**Table 2**).

**Table 2:** Correlations between relative changes of PSM and metabolic indicators during the intervention.

Variables	Total-PSM		Male-PSM		Female-PSM	
	n=130		n=38		n=92	
	r	p-value	r	p-value	r	p-value
FBG	-0.193	0.136	-0.181	0.445	-0.076	0.635
HbA1c	-0.035	0.792	-0.192	0.445	-0.020	0.902
FINS	-0.278	**0.030**	-0.463	**0.040**	-0.216	0.174
HOMO-IR	-0.260	**0.043**	-0.448	**0.048**	-0.191	0.231
TC	-0.206	0.111	-0.309	0.185	-0.280	0.076
TG	-0.312	**0.014**	-0.514	**0.020**	-0.214	0.179
HDL-C	0.243	0.059	0.426	0.061	0.142	0.376
LDL-C	-0.361	**0.004**	-0.499	**0.025**	-0.379	**0.015**
TyG index	-0.333	**0.009**	-0.631	**0.003**	-0.229	0.150
25OHVD	0.080	0.538	0.159	0.503	0.025	0.876
Ca	0.089	0.494	0.245	0.298	0.005	0.975
P	0.070	0.594	0.103	0.666	0.058	0.717
PTH	-0.043	0.747	0.212	0.383	-0.156	0.336
OC	0.383	**0.002**	0.480	**0.032**	0.408	**0.009**

FBG, fasting blood glucose; HbA1c, glycated haemoglobin; FINS, fasting insulin; HOMA-IR, homeostasis model assessment of insulin resistance; TC, total cholesterol; TG, triglycerides; HDL-C, high-density lipoprotein cholesterol; LDL-C, low-density lipoprotein cholesterol; TyG index, triglyceride-glucose index; 25OHVD, 25-OH Vitamin D; Ca, calcium; P, phosphorus; PTH, parathyroid hormone; OC, osteocalcin.

P< 0.05 was considered statistically significant.

Bold values indicate statistically significant.

The data were further analyzed by multiple linear regression based on gender stratification. To mitigate multicollinearity, highly correlated independent variables were excluded. In the male subgroup, FINS, HOMA-IR, TG, and LDL-C were excluded, with the regression model including only the TyG index and OC. In the female subgroup, the regression model encompassed LDL-C and OC. Results indicated that 60.5% of the variations in PSM in males could be elucidated by alterations in OC and the TyG index. Conversely, in females, only 22.7% of PSM changes were explicable by modifications in OC and LDL-C. Nevertheless, upon adjusting for age and BMI, the influence of OC on PSM remained statistically significant solely in the male group (β=0.089, p=0.017) (**Table 3**).

**Table 3:** Multiple linear regression models for the association between relative changes of PSM and metabolic indicators.

Variables	R^2^	β	S.E	Standardized β	95% CI	p-value
Model 1 - Male	0.605					
OC		0.131	0.044	0.455	0.039-0.224	**0.008**
TyG index		-0.459	0.114	-0.612	-0.7-(-0.218)	**0.001**
Model 2 - Female	0.227					
OC		0.059	0.025	0.341	0.008-0.110	**0.024**
LDL-C		-0.076	0.034	-0.327	-0.144-(-0.008)	**0.030**
Model 3 - Male	0.832					
OC		0.089	0.03	0.309	0.019-0.159	**0.017**
TyG index		-0.032	0.128	-0.043	-0.305-0.241	0.805
Model 4 - Female	0.452					
OC		0.040	0.023	0.230	-0.007-0.087	0.092
LDL-C		-0.033	0.032	-0.142	-0.099-0.033	0.313

Dependent variable: PSM.

Model 1/2: Whole sample, stratified by gender.

Model 3/4: Same sample with model 1/2, adjusted for age and BMI.

OC, osteocalcin; TyG index, triglyceride-glucose index; LDL-C, low-density lipoprotein cholesterol.

P< 0.05 was considered statistically significant.

Bold values indicate statistically significant.

## Discussion

### High-protein, energy-restricted diet

Energy-restricted dietary interventions have garnered widespread utilization for weight management among individuals characterized by overweight or obesity. With research substantiating the efficacy of high-protein regimens in averting weight regain after dieting and weight loss (19, 20), we adopted the high-protein, energy-restricted diet in the current study.

Screening for dietary adherence exclusively occurred at the four weekly visits during the initial month of the program. Successful passage through this screening phase enabled subjects to continue the subsequent tri-monthly visits, allowing program continuation even in instances of dietary lapses. Acknowledging the challenges associated with sustained adherence to energy-restricted diets, particularly over prolonged durations, the program’s real-world efficacy is reflected in an average weight loss of 5 kg per participant. Importantly, this weight loss primarily comprises fat reduction, with concurrent preservation of muscle mass. Favorably, glucose and lipid metabolism indicators enhancements were observed compared to pre-intervention values. These findings underscore the merits of endorsing a high-protein, energy-restricted dietary paradigm as a commendable approach to weight loss.

### The effect of OC on muscle mass

Recent years of scientific inquiry have underscored the pivotal role of OC in orchestrating intricate interactions between bone and muscle tissues. Investigations involving older mice have demonstrated that administering OC injections leads to augmented muscle mass, positioning OC as a promising anti-aging agent (6, 7). Human trials further substantiate OC’s involvement in energy metabolism, highlighting its potential to forestall age-related muscle decline. A notable study by Yiting Xu et al. examined 1,742 older adults in Shanghai, revealing a positive correlation between OC levels and the relative skeletal muscle index (SMI, equation: f(x)=SMM(kg)÷body mass(kg)×100 ) (15). Weight-adjusted SMM values were widely acknowledged as a powerful predictor of MetS (21, 22). Following these insights, we adopted a similar approach to convert SMM into PSM, aligning with the percentage of body fat (PBF).

In the aforementioned study by Yiting Xu et al. (15), the relationship between OC and SMI persisted exclusively in hyperglycemic men, suggesting that OC’s protective influence on muscle mass might be mediated indirectly through the amelioration of insulin resistance. In our investigation, subjects with normal FBG were deliberately chosen during recruitment to explore the possibility of a more direct correlation between OC and muscle mass. Despite the absence of MetS development in the subjects, noteworthy enhancements in insulin resistance and lipid profile indicators were observed throughout the weight loss process. Within the male cohort, a correlation emerged between glucose and lipid metabolism indicators and OC with PSM. Further subsequent adjustment for confounding variables revealed that OC maintained an independent association with PSM in men devoid of hyperglycemia, indicative of OC’s direct regulatory impact on muscles.

Contrary to findings in men, the protective effect of OC on muscles did not manifest in women, mirroring observations made by Yiting Xu et al. (15). A plausible explanation resides in the influence of divergent sex hormones. Existing research delineates a positive correlation between testosterone and muscle size and strength in men, while estrogen predominantly affects FM with no or negative implications for muscle mass (23). Consequently, prior investigations on OC have predominantly concentrated on postmenopausal or older women, yielding affirmative outcomes (17, 24, 25). Notably, only 2 out of the 92 female subjects in our study were postmenopausal. Despite the correlation between OC and LDL-C with PSM in the correlation analysis, subsequent regression analysis failed to establish any discernible associations between OC and muscle attributes in women.

### The role of adipose tissue in bone-muscle crosstalk

Adipose tissue (AT) is pivotal in maintaining energy balance, functioning as an endocrine organ that expresses molecules intricately involved in metabolic regulation. A metabolic interrelationship and reciprocal signaling exist between AT and skeletal muscle (SKM) (26–28). Recent studies have highlighted the secretory function of fibro/adipogenic progenitors in governing SKM development and repair (29). In a comprehensive review by Ben Kirk et al., AT and adipokines were identified as the third participants in bone-muscle biochemical crosstalk. The review extensively deliberated on the bidirectional effects of myokines and osteokines on muscle and bone metabolism, along with the influence of adipokines on these secretory organs, modulating bone turnover, BMD, and SKM catabolism (30). In our study, FM was the predominant component of weight loss before and after the program. FM, PBF, and VFA alterations were more significant in the male group than in the female group. The fat reduction is likely to contribute to the protective effect of OC on muscle in men.

Our study has several limitations. Firstly, we solely measured the total OC (tOC) and not the undercarboxylated form (ucOC), which represents the bioactive form and is challenging to quantify. Secondly, the sample size is relatively small, with only 38 male participants included, necessitating adjustments to enhance the study’s representativeness for the broader population. Future research should consider expanding the sample size for a more comprehensive investigation. Additionally, exercise induces a complex endocrine interaction network (31). Earlier investigations have demonstrated that acute exercise can swiftly elevate circulating OC levels (32, 33), and aerobic exercise, akin to resistance exercise, can augment muscle mass (34). In our study, we stipulated a requirement of completing 10,000 steps per day to mitigate these influences. Nevertheless, this measure cannot fully eradicate the impact of exercise on the endocrine network of participants throughout the weight loss process. Subsequent research should integrate more intricate comparative studies to elucidate the dynamic effects of varying exercise intensities on OC and muscle mass.

In summary, upon weight-adjusted data analysis, our study reaffirms the protective impact of OC on SMM during weight loss in men with normal FBG, underscoring a more direct correlation between OC and muscle preservation.

## Data availability statement

The raw data supporting the conclusions of this article will be made available by the authors, without undue reservation.

## Ethics statement

The studies involving humans were approved by Ethics Committee of Xinhua Hospital afliated to Shanghai Jiao Tong University School of Medicine. The studies were conducted in accordance with the local legislation and institutional requirements. The participants provided their written informed consent to participate in this study.

## Author contributions

YX: Data curation, Formal analysis, Methodology, Writing – original draft. WL: Data curation, Investigation, Writing – review & editing. XM: Data curation, Investigation, Writing – review & editing. JZ: Data curation, Investigation, Writing – review & editing. JW: Data curation, Investigation, Writing – review & editing. RX: Supervision, Writing – review & editing. QT: Conceptualization, Supervision, Writing – review & editing.
